# CCR3, CCR5, CCR8 and CXCR3 expression in memory T helper cells from allergic rhinitis patients, asymptomatically sensitized and healthy individuals

**DOI:** 10.1186/1476-7961-4-6

**Published:** 2006-04-19

**Authors:** Mille Holse, Kristian Assing, Lars K Poulsen

**Affiliations:** 1 Laboratory for Medical Allergology 7542, National University Hospital, Blegdamsvej 9, DK-2100 Copenhagen, Denmark

## Abstract

**Background:**

Chemokine receptors have been suggested to be preferentially expressed on CD4+ T cells with CCR3 and CCR8 linked to the T helper (Th) 2 subset and CCR5 and CXCR3 to the Th1 subset, however this remains controversial.

**Objective:**

Our aim was to compare the CCR3, CCR5, CCR8 and CXCR3 expression in memory Th cells from allergic, asymptomatically sensitized and healthy individuals.

**Methods:**

Peripheral blood mononuclear cells from 8 pollen allergic rhinitis patients, 10 asymptomatically sensitized and 10 healthy individuals were stimulated for 7 days with allergen or tetanus toxoid. CCR3, CCR5, CCR8, CXCR3, CD4 and CD45RO were detected by flow cytometry.

**Results:**

No differences in chemokine receptor expression were observed between the three groups on day 0, and seven days of unstimulated culture did not change the expression. Both antigenic stimuli increased the chemokine receptor expression, tetanus toxoid being the most potent. No differences in percentage chemokine receptor positive memory Th cells were observed between the three groups on day 7. Only a change in MFI for CCR5 was significantly different between the three groups after allergen stimulation of the Th cells.

**Conclusion:**

We conclude that even though allergen and antigen induced increased chemokine receptor expression, no differences in profiles were identified in memory Th cells from patient groups with different atopic status.

## Introduction

The prevalence of allergy is increasing in the westernized part of the world with estimates suggesting that 20–30% of the population is affected [1]. However, unlike the reaction of most IgE-sensitized individuals who upon re-exposure to the allergen develop symptoms due to activation and release of mediators from various immune cells, some individuals seem to exhibit an IgE positive phenotype without having any allergic symptoms. These individuals have been described in the literature as *asymptomatically sensitized *and are phenotypically considered to be a group between the allergic and the healthy individuals with an increased risk of developing allergy [2,3].

The chemokines and their receptors play a pivotal role in leukocyte migration and chemotaxis. It is still controversial whether these receptors can function as phenotypic markers on certain cell subsets. CCR3 and CCR8 have been suggested as Th2 markers whereas CXCR3 is mentioned in the literature as a Th1 marker [4-6]. The CCR3 ligand CCL11/eotaxin is upregulated in nasal mucosa of allergic rhinitis patients during the pollen season [7]. CCL1/I-309, which is the only CCR8 ligand, is upregulated in patients with atopic dermatitis [8] and IL-12 inhibit its production [9]. Also, CCL1/I-309 is released by mast cells in response to IgE cross-linking [10] indicating a role in allergic inflammation. On the contrary, the IFN-γ-inducible CXCR3 ligands and some CCR5 ligands are increased in autoimmune diseases [11-14]. However, other findings show that no correlation exists between Th1/Th2 cytokine profile and chemokine receptor expression on a single cell level [15] and also suggest that the chemokine receptor profile can be changed without a concomitant change in cytokine profile [16] questioning the use of chemokine receptors as markers for T cell subsets.

As the chemokine receptor profile determines the migratory patterns of leukocytes, we wanted to compare this profile with respect to CCR3, CCR5, CCR8 and CXCR3 in memory Th cells from allergic, asymptomatically sensitized and healthy individuals to obtain knowledge about their migratory potential and any differences in expression patterns that might exist between these three groups.

## Methods

### Patients

10 healthy, 5 asymptomatically birch pollen sensitized, 5 asymptomatically grass pollen sensitized, 5 birch pollen allergic and 3 grass pollen allergic volunteers with seasonal hay fever symptoms were examined during the birch or grass pollen season respectively. Skin prick test (Soluprick, ALK-Abello, Hørsholm, Copenhagen), histamine release [17] and specific IgE against birch and grass pollen using the CAP-system (Pharmacia, Uppsala, Sweden) were determined for all volunteers (Table 1). The skin prick test was performed according to the guidelines of European Academy of Allergy and Clinical Immunology [18]. Three of the allergic patients had allergic asthma. The allergic subjects received no corticosteroid treatment for three months prior to the study. The asymptomatically sensitized and healthy control subjects took no hay fever medicine. All subjects came from the area of greater Copenhagen (Storkøbenhavn). The study was approved by the local Ethical Committee and the clinical features of the patients are described in detail elsewhere [19].

**Table 1:** Patient characterization.

			**Birch**				**Grass**			
**Patients**	**Age** (range)	**Sex** Male/Female	Symptoms	Spt	IgE class Median(range)	HR class Median(range)	Symptoms	Spt	IgE class Median(range)	HR class Median(range)

Healthy	25 (22–43)	3/7	0/10	0/10	0 (0)	0 (0)	0/10	0/10	0 (0)	0 (0)
AS Birch	25 (24–27)	1/4	0/5	5/5	0 (0–2)	2 (0–3)	0/5	0/5	0 (0)	0 (0)
AS Grass	25 (22–31)	1/4	0/5	0/5	0 (0)	0 (0)	0/5	5/5	0 (0–2)	0 (0–3)
Allergic Birch	27 (25–43)	4/1	5/5	5/5	3 (2–4)	3 (2–3)	2/5	2/5	0 (0–4)	2 (0–3)
Allergic Grass	26 (24–41)	3/0	1/3	1/3	0 (0–3)	0 (0–3)	3/3	3/3	4 (2–4)	3 (0–3)

AS: asymptomatically sensitized. Spt: skin prick test. HR: histamine release

Skin prick tests (performed in duplicate) were considered positive when mean wheal diameter >3 mm. Allergic symptoms were reported in diary cards during the relevant pollen season. Symptoms were considered as pollen allergy when lasting > 7 days or when symptoms were repeatedly elicited when pollen counts exceeded a certain (individual) level [2]. The skin prick test was performed according to the guidelines of European Academy of Allergy and Clinical Immunology [18]. n = 10 for the healthy controls, n = 10 for the asymptomatically sensitized individuals and n = 8 for the allergic individuals.

### Cell stimulation

Peripheral blood mononuclear cells (PBMCs) were isolated from whole blood by gradient centrifugation on Lymphoprep (Nycomed, Roskilde, Denmark). The PBMCs were cultured (3 × 10^6^) in 6 well plates with antigen in 6 ml of low endotoxin RPMI1640 medium containing 10% heat-inactivated autologous serum, 25 mM HEPES, 2 mM L-glutamine, 50 μM β-mercaptoethanol, 100 U/ml streptomycin/penicillin for 7 days in the presence of either 15 μg/ml birch or grass allergen (ALK-Abello, Hørsholm, Copenhagen), 10 μg/ml Tetanus toxoid (TTx) (Statens Serum Institut, Copenhagen, Denmark) or no antigen as a control. On day 7, the cells were harvested and used for flow cytometric analysis. The lipopolysaccharide level in both allergen extracts was < 7 EU/mg and after 24 hours of stimulation of PBMCs from healthy individuals no detectable amounts of TNF-α were observed.

### Flow cytometry

Surface markers were detected using primary labeled antibodies: CCR3-FITC, CCR5-FITC, CCR8-FITC, CXCR3-FITC (R&Dsystems, Abingdon, UK), CD4-PE-Cy5 and CD45RO-PE (Dakocytomation, Glostrup, Denmark). Three-color flow cytometry was performed on day 0 and day 7 on a FACScan (Becton Dickinson, Heidelberg, Germany) using WinList (Verity Software House, Topsham, USA) software for analysis. Isotype control cut-off values were set to > 98% and 10.000 PBMC were acquired. Gating was done by firstly applying a CD4+ gate followed by determination of the percentage and mean fluorescence intensity (MFI) of the CD45RO and chemokine receptor double positive population. CD45RO is a marker of effector and memory T cells, however throughout this article these CD4+ CD45RO+ T cells will be mentioned as memory T helper cells for convenience.

### Statistical analysis

Samples were compared using non-parametric statistics (Kruskall-Wallis test or Wilcoxon's test for matched pairs). Values of P < 0.05 were considered significant.

## Results

### Day 0

**Figure 1:**
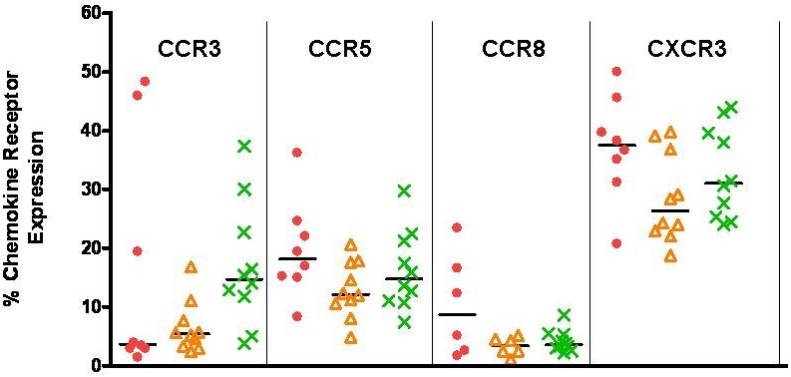
**Percentage chemokine receptor positive memory T helper cells day 0**. Percentage CCR3+, CCR5+, CCR8+ and CXCR3+ memory Th cells from allergic (dots), asymptomatically sensitized (triangles) and healthy control (crosses) individuals on day 0 immediate *ex vivo*. - = median value. n = 10 for the healthy controls, n = 10 for the asymptomatically sensitized and n = 8 for the allergic individuals except for CCR8 where n = 6 for the asymptomatically sensitized and allergic individuals. 10.000 PBMCs were acquired for the analysis. Isotype control cut-off values were set to >98%. Samples were run in monocates. For experimental design and analysis see Methods.

Immediate after isolation of the PBMCs, the cells were subjected to flow cytometric analysis. No significant differences in the percentage of CCR3+, CCR5+, CCR8+ and CXCR3+ memory Th cells from allergic, asymptomatically sensitized and healthy individuals were observed (Figure 1). Likewise, no differences in MFI were observed between the three donor groups (results not shown).

### Day 7

#### Effect of stimulation

No significant differences in chemokine receptors (neither expressed as the percentage of positive cells nor as MFI) were observed between day 0 and the cells having been kept in antigen-free medium for 7 days as controls. Thus the medium alone and the experimental set-up did not influence the chemokine receptor expression. After TTx stimulation a significant increase in MFI was observed for CCR3 in the allergic and asymptomatically sensitized individuals, but not in the healthy control group (Table 2). However, no increases in the percentage of CCR3+ memory Th cells were observed in any of the groups. CCR5 increased both as MFI and the percentage of CCR5+ memory Th cells in the asymptomatically sensitized and healthy control group whereas no changes in CCR5 was observed in the allergic individuals. Also, an increase in the percentage of CCR8+ memory Th cells was observed in the healthy control group, but no significant changes in MFI were observed for this receptor. An increase in the percentage of CXCR3+ memory Th cells was observed in all three groups after TTx stimulation, however increases in MFI were only observed in the healthy control group.

**Table 2:** Chemokine receptor expression in memory T helper cells induced by 7 days of antigenic stimuli. Median chemokine receptor expression in memory Th cells in percentage and MFI after 7 days of stimulation with antigen (allergen (15 μg/ml) or TTx (10 μg/ml)) or no antigen as a control. n = 10 for the healthy controls, n = 10 for the asymptomatically sensitized individuals and n = 8 for the allergic individuals except for CCR8 where n = 6 for the asymptomatically sensitized and allergic individuals. 10.000 PBMCs were acquired for the analysis. Isotype control cut-off values were set to >98%. Samples were run in monocates. For experimental design and analysis see Methods.

		% median (range)			MFI median (range)		
		***No Ag***	**Allergen**	**TTx**	***No Ag***	**Allergen**	**TTx**

**CCR3**	Allergic	*10.6 (4.7–32)*	17.3 (9.8–29.5)	15.6 (4.4–52.2)	*21 (16.2–32.3)*	19.4 (16.9–37.2)	26.1* (16.6–40.5)
	AS	*10.3 (3.3–19.7)*	8.7 (2.8–23.2)	11.9 (5–28.8)	*20.5 (14.7–27.2)*	18.6 (16.4–32.3)	23.4 * (18.4–37.4)
	Healthy	*8.7 (1.5–45)*	11.2 (3.2–36.8)	23.4 (4.5–44.3)	*19.7 (12.6–45.4)*	17.8 (12.5–32.2)	23.7 (12.5–38.4)
**CCR5**	Allergic	*21.3 (7.8–31.5)*	25 (4.1–53.9)	28.3 (12.3–61.1)	*21.4 (16.8–27.9)*	25.9 * (22.4–48.5)	26.1 (16.1–42.5)
	AS	*15.2 (2.6–36.6)*	17 (3–33.5)	16.9 * (5.2–65.9)	*18.3 (15.2–24.5)*	17.5 (15.4–20.5)	22.5 * (18.2–58.2)
	Healthy	*11.7 (5.6–34.5)*	13.5 * (6.7–29.7)	20.2 * (10.1–74.1)	*16.7 (12.7–22)*	17.3 (13.5–25.6)	25.1 * (13.4–72)
**CCR8**	Allergic	*10.4 (1.6–20.4)*	12.8 (3.3–20)	12.7 (2.2–27.4)	*24.3 (16.8–37.6)*	27.9 (18.8–40.3)	28.2 (17.8–82.6)
	AS	*3.7 (1.8–16.6)*	3.2 (1.7–14.7)	6.3 (1.4–34.9)	*21.9 (17–26.1)*	22.8 (17.2–44.6)	24.9 (16.2–36.6)
	Healthy	*5.7 (0.7–16.4)*	3.9 (1.5–14)	9.3 * (3.8–35.7)	*19 (12.6–49.6)*	19.2 (11.7–46.6)	23.9 (12.4–48.2)
**CXCR3**	Allergic	*42.9 (22–47.5)*	42.1 (19.2–67.4)	49.2 * (27–74.2)	*57.4 (33.1–65.3)*	54.4 (48.4–72.4)	60.6 (36.9–81.8)
	AS	*28.1 (18–56.6)*	27.8 (21–56.8)	29 * (20.8–77.4)	*39.7 (29.3–59.7)*	42.3 (30.6–54.5)	46.5 (33.5–115)
	Healthy	*28.8 (19.8–46.5)*	31.4 (17.3–46.9)	35.9 * (25.8–82.9)	*37.8 (32–65.2)*	39.3 (30.1–63.9)	57.6 * (32–162.4)

Bold text and * indicates significant differences between the antigen (allergen or TTx) stimulated samples and control samples where no antigen was added.

Ag: antigen AS: asymptomatically sensitized individuals.

After stimulation with allergen, increases in the percentage of CCR5+ memory Th cells were observed in healthy controls and in MFI in allergic individuals. Allergen stimulation did not induce any changes in CCR3, CCR8 and CXCR3 expression. When pooling all 28 patients in the statistical analysis, TTx was able to induce expression of all receptors both seen as a significant increase in the percentage of chemokine receptor positive cells and as MFI. Allergen stimulation only induced a significant increase in the percentage of CCR5+ memory Th cells.

#### Group differences

To compare the chemokine receptor expression between the three groups, the Day 7_no antigen _receptor level was subtracted from either the Day 7_allergen _or the Day 7_TTx _sample to obtain the change in receptor expression (ΔChemokine receptor).

No differences in ΔChemokine receptor for the percentage of chemokine receptor positive cells were observed between the three groups after stimulation with TTx or allergen. When comparing the ΔChemokine receptor for the MFI, the change in the CCR5 after allergen stimulation was significantly different between the three groups (P = 0.02).

## Discussion

Other studies have linked certain diseases with aberrant expression of one or more chemokine receptors [12,20,21]. However, very few studies have been conducted with regard to the phenotype of asymptomatically sensitized individuals and, to our knowledge none on chemokine receptor profiles.

In this study, no differences were found in receptor expression patterns immediate *ex vivo *for CCR3, CCR5, CCR8 and CXCR3 in memory Th cells from allergic, asymptomatically sensitized and healthy individuals despite the fact that the study was carried out in the pollen season.

Our findings are in agreement with other studies reporting equal mRNA levels of CCR3 and CCR5 in PBMCs [22] and same levels of CXCR3+ peripheral blood Th cells [21] in patients with atopic dermatitis and healthy controls, but in disagreement with other findings showing decreased percentage of CCR5+ and CXCR3+ memory Th cells in the blood from patients with atopic dermatitis compared to healthy controls [23].

Changes in chemokine receptor expression were observed after stimulation with both antigens (Table 2). CCR5 expression was induced after TTx stimulation, but only in asymptomatically sensitized and healthy individuals. The reason why allergic individuals do not upregulate this receptor even when stimulated with a type 1 antigen is speculative, but one reason might be due to their Th2 biased reaction pathway. However, they do show significant increases in percentage CXCR3+ memory Th cells after TTx stimulation, in accordance with this receptor's much stronger link to the Th1 phenotype [24].

Only when grouping all individuals, did the recall antigen TTx induce significant increases in expression of all receptors. The reason for the less clear effect as observed in the individual groups might be due to the great inter-individual variation in chemokine receptor expression level, an observation also described by others [25]. Nevertheless, TTx induced more changes than the allergenic stimuli, an effect that is likely due to the higher frequency of TTx specific T cells compared to allergen specific T cells in peripheral blood (Glue, unpublished results).

CCR5 appears to be the most allergen susceptible receptor. However, the apparent overlap in expression levels between the groups would exclude the use of this receptor as a diagnostic tool and thus is of no major clinical interest.

When comparing the three groups after antigen stimulation, we found no differences in expression patterns between the three groups except for the change in CCR5 MFI which was significantly different between the three groups. This is the only observed difference between the three groups but as discussed above the great overlap in receptor levels would not make this finding of any clinical relevance.

In spite of the apparent lack of differences between the three groups with respect to chemokine receptor profile, a parallel study using a somewhat larger sample size showed that allergen stimulation induced significantly more proliferation of memory Th cells in the allergic individuals compared to the asymptomatically sensitized and healthy individuals as well as a different cytokine profile [19].

## Conclusion

In conclusion, both antigenic stimuli were able to induce changes in chemokine receptor expression. TTx seemed to be a more potent stimulus with regard to changes in chemokine receptor expression in all three groups compared to the pollen allergens. No major differences in CCR3, CCR5, CCR8 and CXCR3 were found between allergic, asymptomatically sensitized and healthy individuals and thus chemokine receptor expression in peripheral blood memory Th cells does not seem to be linked to patient status. No major differences were seen between the three groups after antigenic stimulation and thus we conclude that pollen allergic, asymptomatically pollen sensitized and healthy individuals cannot be distinguished by means of chemokine receptors expression in memory Th cells and thus the migratory potentials of the memory Th cells seem to be the same between the three groups.

## Abbreviations

Ag: antigen AS: asymptomatically sensitized MFI: mean fluorescence intensity PBMC: peripheral blood mononuclear cell Th: T helper TTx: Tetanus toxoid

## Competing interests

The author(s) declare that they have no competing interests.

## Authors' contributions

All authors participated in the design of the study. KA conducted the patient contact and characterization, cell isolation and stimulation assays. MH conducted the flow cytometry and analyzed the data. All authors contributed towards the manuscript preparation with MH as the main author of the article.
